# Genetically Predicted Testosterone and Systemic Inflammation in Men: A Separate-Sample Mendelian Randomization Analysis in Older Chinese Men

**DOI:** 10.1371/journal.pone.0126442

**Published:** 2015-05-07

**Authors:** Jie Zhao, Chaoqiang Jiang, Tai Hing Lam, Bin Liu, Kar Keung Cheng, Lin Xu, Shiu Lun Au Yeung, Weisen Zhang, Gabriel M. Leung, C. Mary Schooling

**Affiliations:** 1 School of Public Health, Li Ka Shing Faculty of Medicine, The University of Hong Kong, Hong Kong Special Administrative Region, Hong Kong, China; 2 Guangzhou Number 12 Hospital, Guangzhou, China; 3 Department of Public Health and Epidemiology, University of Birmingham, Birmingham, United Kingdom; 4 School of Urban Public Health, Hunter College, CUNY School of Public Health, New York, New York, United States of America; University of Sydney, AUSTRALIA

## Abstract

**Objectives:**

Observationally, testosterone is negatively associated with systemic inflammation, but this association is open to both residual confounding and reverse causality. Large-scale randomized controlled trials (RCTs), assessing exogenous effects, are presently unavailable. We examined the association of endogenous testosterone with well-established systemic inflammatory markers (white blood cell, granulocyte, lymphocyte and high-sensitivity C-reactive protein (hsCRP)) using a separate-sample Mendelian randomization analysis to minimize reverse causality.

**Methods:**

A genetic prediction rule for serum testosterone was developed in 289 young Chinese men with mean age of 21.0, using selected testosterone-related SNPs (rs10046, rs1008805 and rs1256031). Multivariable linear regression was used to examine the association of genetically predicted serum testosterone with inflammatory markers among 4,212 older Chinese men from the Guangzhou Biobank Cohort Study.

**Results:**

Genetically predicted testosterone was unrelated to white blood cell count (-0.01 109/L per nmol/L testosterone, 95% confidence interval (CI) -0.05 to 0.04), granulocyte count (-0.02 109/L, 95% CI -0.06 to 0.02), lymphocyte count (0.005 109/L, 95% CI -0.01 to 0.02) and hsCRP (-0.05 mg/L, 95% CI -0.15 to 0.06).

**Conclusion:**

Our findings did not corroborate any anti-inflammatory effects of testosterone or corresponding potentially protective effects of testosterone on chronic diseases resulting from reduced low-grade systemic inflammation.

## Introduction

Inflammation is an important component of major non-communicable diseases, such as cardiovascular disease (CVD) and diabetes [1]. The low-grade systemic inflammation characterizing CVD and other chronic diseases is not well understood, but appears to be a state of immune dysregulation or dysfunction [1]. Since the 1920s, when testicular extracts were first developed, androgens have been known to affect aspects of the immune system in animals [2,3]. White blood cell (WBC) count and high-sensitivity C-reactive protein (hsCRP) are well-established markers of systemic inflammation and also predictive for CVD mortality [4,5]. Most, but not all, observational studies report testosterone inversely associated with C-reactive protein (CRP) [6,7] and WBC and/or its differentials [8,9]. However, these study designs are open to residual confounding and reverse causality [10] arising from testosterone falling with age and the effects of ill-health [11,12]. Small-scale randomized controlled trials (RCTs), designed to assess other outcomes, generally report no effect of testosterone administration on CRP [13–15] or WBC [16], although one RCT among hypogonadal men with metabolic syndrome found testosterone decreased CRP [16]. To our knowledge, there is no material evidence from meta-analysis of RCTs or large-scale RCTs.

A Mendelian randomization (MR) design, using testosterone related genetic variants as an instrumental variable, provides a means of assessing the causal effect of testosterone on markers of systemic inflammation without any intervention. Genetic variants, resulting in life-long differences in endogenous exposures, are determined at conception, and so are unlikely to be associated with socioeconomic position or other confounders. Genetic polymorphisms affect testosterone [17], making an MR design feasible. Using an MR analysis with a separate-sample instrumental variable (SSIV) estimator, we examined the association of testosterone in early adulthood, when testosterone levels are at their lifetime peak, with well-established markers of systemic inflammation among men.

## Materials and Methods

### Study design

This study uses a separate-sample two-stage MR analysis. First, a genetic prediction rule for serum testosterone was developed in young Chinese men from Hong Kong, as described previously [18]. Second, the association of genetically predicted testosterone with WBC, lymphocyte and granulocyte counts and hsCRP among older Chinese men from the Guangzhou Biobank Cohort Study (GBCS) was examined [19].

### Sources of data

To generate a genetic prediction rule for testosterone, a sample of young men with parents and at least three grandparents born in Hong Kong or Guangdong province was recruited, but restricted to those not taking any medication potentially affecting testosterone. Morning blood samples were taken for testosterone assessment and DNA extraction. Testosterone was assessed by competitive immunoassay on Vitros 3600 immunodiagnostic system (Ortho Clinical Diagnostics Inc, USA) with a detection limit of 0.17 nmol/L. Calibrators for this assay were in-house reference materials from Ortho Clinical Diagnostics that were value-assigned to correlate to samples measured by isotope-dilution gas chromatography-mass spectroscopy (ID-GC/MS). The intra- and inter-assay coefficients of variation (CVs) were 4.9% and 5.7% at 4.4 nmol/L, 3.2% and 3.9% at 16.3 nmol/L, and 1.8% and 3.0% at 37.5 nmol/L, respectively. DNA was extracted and analyzed at the Centre for Genomic Sciences of the University of Hong Kong for selected SNPs, selected as shown in S1 Fig, including SNPs from *ESR1* (rs722208 and rs2175898), *CYP19A1* (rs10046 and rs1008805) and *ESR2* (rs1256030 and rs1256031) using a Mass ARRAY system (Sequenom, San Diego, California), as described previously [18]. For DNA quality analysis we used spectrophotometry for most of the samples and gel electrophoresis for 4 duplicate check controls and 6 randomly selected samples in each DNA sample plate. The determined sample concentration and A260/280 ratios were 10–20 ng/μL and 1.7–2.0 respectively. A call rate <80% was considered failure. All the SNPs passed with a call rate >95%. A self-administered questionnaire was used to collect socioeconomic position and health status.

To examine the association of genetically predicted testosterone with markers of inflammation, we used a large sample of older men (50+ years) from GBCS. GBCS is an ongoing collaboration of Guangzhou Number 12 Hospital, the Universities of Hong Kong and Birmingham, UK, as described elsewhere [19]. All participants were permanent residents of Guangzhou and members of the “The Guangzhou Health and Happiness Association for the Respectable Elders” (GHHARE), a community social and welfare association unofficially aligned with the municipal government. Membership is open to older people for a monthly fee of 4 Yuan (50 US cents). Recruitment occurred in 3 phases: phase 1 from September 2003 to November 2004, phase 2 from April 2005 to May 2006, and phase 3 from September 2006 to January 2008. Follow-up of the participants started in 2008. About 7% of permanent Guangzhou residents aged 50+ years are members of GHHARE, of whom 11% (about 10,000 participants) enrolled for each of phases one, two and three. Inclusion criteria were that they were capable of consenting, that they were ambulatory, and that they were not receiving treatment modalities which if omitted may result in immediate life threatening risk, such as chemotherapy or radiotherapy for cancer, or dialysis for renal failure. Fasting blood samples were collected at recruitment in phase 3 and at follow-up for participants recruited in other phases. Samples were stored, as whole blood or as buffy coat and sera, at -80°C [19] for all apart from a subset of phase 3 participants whose DNA was extracted from fresh blood and stored at -80°C [20]. The detailed methods of measurement have been reported previously [19,21,22]. Quantitative haematological analysis was performed using a SYSMEX KX-21 haematology analyser, from which white blood cell count, lymphocyte cell count and granulocyte cell count were available. HsCRP was determined by the Shimadzu CL-8000 Clinical Chemical Analyser (Shimadzu Corp, Kyoto, Japan) in the hospital laboratory. Selected SNPs were analyzed by a commercial company (Beijing CapitalBio Corporation) in Beijing using a Mass ARRAY system (Sequenom, San Diego, California). Any measurement error is likely to be non-differential, which is compensated for by our large sample size. The University of Hong Kong-Hospital Authority Hong Kong West Cluster Joint Institutional Review Board approved the study. The Guangzhou Medical Ethics Committee of the Chinese Medical Association approved GBCS, including the use of genetic data, and all participants gave written, informed consent prior to participation.

### Exposure

The primary exposure was genetically predicted testosterone. Genetically predicted log testosterone was obtained from stepwise regression of all selected SNPs on testosterone in the sample of young men, which gave predicted log testosterone as *-0*.*07×rs1008805+0*.*07×rs10046-0*.*07×rs1256031+3*.*0*, as in S1 Table and as reported previously [18]. The F-statistic was 13.3, suggesting a reliable genetic instrument, because an F-statistic>10 suggests a reliable genetic instrument [23]. The proportion of variance in log testosterone explained by the genetic prediction rule was 4.1%, which is typical for MR studies [20]. Higher genetically predicted log testosterone was associated with higher log serum testosterone. Genetically predicted testosterone was estimated as the anti-log of genetically predicted log testosterone. Testosterone, instead of log testosterone, was used as the exposure for ease of interpretation as they both gave the same pattern of results.

### Outcomes

The primary outcomes were inflammatory markers including WBC count, lymphocyte cell count, granulocyte cell count and hsCRP.

### Statistical analysis

We used an SSIV estimate from two separate samples [24]. ANOVA was used to compare genetically predicted testosterone by key characteristics. Multivariable linear regression was used to assess the association of genetically predicted testosterone with markers of systemic inflammation. Power calculation was conducted using the formulas in the study of Freeman et al. [25], and compared with the simulation results in the study of Pierce et al [26]. All statistical analyses were conducted using Stata 10.1 (StataCorp LP, College Station, Texas).

## Results

Among the 8,450 men in all 3 phases of GBCS, DNA for SNP testing was available for 4,262 men; availability depended on recruitment phase and other logistical concerns, but not on inflammatory status. Among the 4,262 men, 4,212 (98.8%) had complete information on the selected SNPs. However, hsCRP was only measured in phase 1 and was only available for 1,636 men.

Table 1 shows, as expected, genetically predicted testosterone among the older men was not associated with age, education, smoking and use of alcohol. Genetically predicted testosterone (mean = 18.0 nmol/L, standard deviation = 1.2 nmol/L) does not capture variation due to confounders.

**Table 1:** Genetically Predicted Testosterone by Socio-demographic Characteristics Among 4,212 Men (50+), Guangzhou Biobank Cohort Study, 2003–2008.

Characteristic	No.	%	Genetically predicted testosterone (nmol/L)		
			Mean	SD	ANOVA *p* value^a^
Age group, years	4212				0.94
50–54		8.1	18.05	1.10	
55–59		20.2	18.01	1.22	
60–64		25.4	18.01	1.24	
65–69		24.1	18.01	1.27	
70–74		16.5	17.99	1.19	
75–79		4.2	17.95	1.28	
≥80		1.5	18.15	1.52	
Education	4209				0.91
Less than primary school		2.6	18.11	1.36	
Primary school		26.9	18.01	1.22	
Junior middle school		30.0	18.01	1.19	
Senior middle school		24.2	18.01	1.23	
Junior college		8.9	17.99	1.34	
College		7.4	17.96	1.24	
Smoking status	4192				0.59
Never smoker		40.8	17.99	1.22	
Ex-smoker		27.9	18.02	1.25	
Current smoker		31.3	18.03	1.22	
Use of alcohol	4212				0.49
Never		51.8	18.02	1.25	
<1/month		19.9	18.03	1.19	
<1/week		4.3	18.04	1.20	
1-4/week		6.0	18.03	1.30	
5+/week		11.9	17.90	1.19	
Ex-drinker		4.4	18.01	1.20	
Unknown		1.8	18.08	1.27	

Abbreviation: SD, standard deviation

^a^ Genetically predicted testosterone was not associated with age, socioeconomic position (education) or lifestyle, including smoking status and use of alcohol (ANOVA *p* value>0.05).

Table 2 shows genetically predicted testosterone among the older men was not associated with markers of systemic inflammation. The estimates were close to the null.

**Table 2:** Effect of genetically predicted testosterone on markers of systemic inflammation among 4182 and a subgroup of 1636 men (50+) in a Mendelian randomization study design, Guangzhou Biobank Cohort Study, 2003–2008.

Outcome	n	Beta coefficient^a^	95% CI
WBC Count	4180	-0.01	-0.05 to 0.04
(10^9^/L)			
Granulocyte	4167	-0.02	-0.06 to 0.02
(10^9^/L)			
Lymphocyte	4182	0.005	-0.01 to 0.02
(10^9^/L)			
hsCRP (mg/L)	1636	-0.05	-0.15 to 0.06

^a^Beta coefficient refers to the average change in white blood cell and its differentials counts and hsCRP level with each unit (nmol/L) increase in genetically predicted testosterone.

## Discussion

Using a separate-sample MR analysis in Chinese men, we found no association of endogenous testosterone with markers of systemic inflammation. Our novel study provides no support for any anti-inflammatory effects of testosterone on hsCRP, WBC or its differentials, although we cannot preclude possible effects of testosterone for other inflammatory markers. Our study from China also adds to the limited findings from developing countries in contrast to most previous studies from developed countries.

To our knowledge, this is the first study to use an MR design, with an SSIV estimator, to provide an unbiased estimate of the effects of genetically predicted testosterone on systemic inflammation. In cross-sectional studies, it is difficult to determine causal direction because some markers of systemic inflammation, such as pro-inflammatory cytokines, inhibit testosterone secretion through their influence on the hypothalamic-pituitary-gonadotropic axis [27]. However, our study using genetically predicted testosterone in early adulthood minimizes such reverse causality and provides stronger evidence with minimal confounding. Compared with traditional IV analysis, SSIV is more useful and cost-efficient when the phenotype of interest is either not measured or is measured with substantial error in the sample with the outcome [24,28]. Simulations have shown SSIV is preferable to using the same sample for assessing the relations of instrument to exposure and instrument to outcome [29]. The SSIV design also makes it feasible to use testosterone in early adulthood as a marker of lifetime exposure when it is infeasible to obtain lifetime sex hormones for the older people, because it would require measurements from the 1960s. SSIV remediates weak-instruments bias and reduces concerns about using multiple polymorphisms as IVs [24]. In addition, any correlation of the genetic variants with unmeasured confounders in the sample with the phenotype is unlikely to be replicated in the sample with the outcome due to the different data structures [24].

Our findings are consistent with most RCTs reporting no significant effects of testosterone therapy on markers of systemic inflammation [13–16]. Our results are less consistent with observational studies [6–9], where testosterone tends to be negatively associated with markers of systemic inflammation. This study does not corroborate predictions from an evolutionary biology perspective, where androgens might be expected to be positively associated with systemic inflammatory markers as a reflection of their immunosuppressive role [30]. Several possible explanations exist. First, serum testosterone may reflect androgen production more than androgen activity. Successful use of anti-androgens at castrate levels of serum testosterone in prostate cancer trials has challenged the extent to which serum testosterone reflects androgen activity [31]. Serum testosterone is only weakly correlated with the final metabolite of all androgens [32,33]. Second, androgens may be positively related with other markers of systemic inflammation, such as soluble IL-6 and TNF-alpha. However, hsCRP is closely correlated with IL-6 and TNF-alpha, and gives consistent associations with CVD risk factors [34].

Although we used an MR analysis design with similar properties to an RCT [10], limitations exist. First, two independent samples were used and genetic associations derived from one sample might not apply to the other. However, the two samples had the same genetic origin, because residents of Hong Kong and Guangzhou (the capital of Guangdong province) share a recent, common ancestry, as the Hong Kong population was largely formed by migration from Guangdong province in the late 1940s and early 1950s [35]. We restricted the sample of young men to those with both parents and at least 3 grandparents born in Hong Kong or Guangdong province to ensure genetic homogeneity, reflected by the similar allele frequencies of the genetic variants in the two samples [18]. Moreover, it is generally assumed in genetic studies that a gene (and hence) a genetic score has the same effect on the phenotype across time and space. In the same study, genetically predicted testosterone had the expected negative relation with HDL-cholesterol [18], of the expected order of magnitude [36], also suggesting some validity. Second, the effect of testosterone on markers of systemic inflammation might be confounded by other sex hormones. However, estrogens are not thought to be converted to testosterone in men [37], and so do not meet the criteria of being a confounder [10]. Third, population stratification and canalization may affect MR analysis. However, the participants were restricted to ethnic Chinese men with no known canalization, i.e., compensatory adaptation to genetic effects during development [10]. Fourth, MR is based on the assumption that the genetic instrument is associated with the exposure, is not associated with the outcome other than via the exposure (no pleiotropy exists) and no confounders of the association of the genetic instrument with the outcomes exist [38]. We used SNPs from genes functionally relevant to testosterone. The selected SNPs may affect systemic inflammation directly (pleiotropy) rather than via testosterone, however, no such evidence could be identified. The genetic prediction rule was not associated with age, education or lifestyle (Table 1). Fifth, testosterone predicted from the genetic prediction rule is different from measured serum testosterone, as the genetic instrument only explains a small proportion of the variance. Therefore, the estimates from MR are less precise (although less biased) than those from traditional regression [10]. MR studies require a large sample size: approximately the usual sample size for exposure on outcome divided by the R^2^ (here 0.04) between instrument and exposure [25]. The power of separate-sample MR is approximately equal to the power of traditional complete-data analysis in the traditional MR setting [28]. According to the formulas in the study of Freeman et al [25], for WBC, granulocyte and lymphocyte counts, a sample size of ~4,200 had 0.8 power to detect a relatively small effect size of 0.22, although the analysis of hsCRP, with a sample size of ~1,600 only had 0.8 power to detect an effect size of 0.35. This is also consistent with simulation results from Pierce et al [26]. The formulas provide a relatively simple and fast way to approximate the sample size required for a specific sample size to achieve a fixed power [25]. In GBCS, an effect size of 0.22 is equivalent to a difference of about 0.37 10^9^/L in WBC count, 0.34 10^9^/L in granulocyte count, and 0.14 10^9^/L in lymphocyte count. Differences smaller than these are not expected to be clinically meaningful, although could make a difference on a population level. We used a weighted genetic prediction rule instead of single SNP as instrument, reducing variability in MR estimation [39]. Replication with a stronger instrument in a larger sample or meta-analysis of MR analyses is needed.

## Conclusions

The study did not corroborate observations of an association of endogenous testosterone with lower levels of markers of systemic inflammation and correspondingly any potentially protective effects of testosterone on complex chronic diseases via reducing low-grade systemic inflammation.

## Supporting Information

S1 Table Genotypes of selected SNPs and genetic associations with log testosterone in the young men. (DOCX)

S1 Fig Flow chart for SNP selection. For references identified in the figure, please see Appendix references in S1 File.
(TIF)

S1 File Appendix references. (DOCX)
