# Supplementary material for: Genetically Predicted Testosterone and Systemic Inflammation in Men: A Separate-Sample Mendelian Randomization Analysis in Older Chinese Men
Source: PLoS One. 2015 May 7;10(5):e0126442. doi: 10.1371/journal.pone.0126442 (PMC4423952; doi:10.1371/journal.pone.0126442)
Supplement: S1 Table — (DOCX) [file pone.0126442.s001.docx]

**S1 Table.** Genotypes of selected SNPs and genetic associations with log testosterone in the young men

| Gene | SNP | Genotype^a^ | Genetic associations with log testosterone (nmol/L)^b^ | | |
| --- | --- | --- | --- | --- | --- |
|  |  |  | Beta-coefficient | 95% CI | *P* value |
|  |  |  |  |  |  |
| *CYP19A1* | rs1008805 | CC, CT, TT | -0.07 | -0.14, -0.01 | 0.03 |
|  |  |  |  |  |  |
| *CYP19A1* | rs10046 | CC, CT, TT | 0.07 | 0.01, 0.13 | 0.02 |
|  |  |  |  |  |  |
| *ESR2* | rs1256031 | CC, CT, TT | -0.07 | -0.12, -0.02 | 0.01 |
|  |  |  |  |  |  |

^a^ Genotypes of CC, CT and TT were coded with 0, 1, and 2 respectively in the regression model.

^b^ In the regression model, log testosterone was used as outcome, because the distribution of testosterone was skewed. Two outliers (Cook’D value>0.05) were dropped when establishing the genetic prediction rule for log testosterone, so 287 men were included in the prediction model.
