# Supplementary figures and images for: Genetically Predicted Testosterone and Systemic Inflammation in Men: A Separate-Sample Mendelian Randomization Analysis in Older Chinese Men
Source: PLoS One. 2015 May 7;10(5):e0126442. doi: 10.1371/journal.pone.0126442 (PMC4423952; doi:10.1371/journal.pone.0126442)

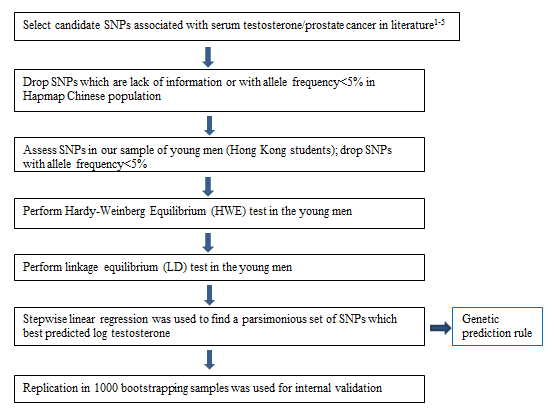

Supplement: S1 Fig — For references identified in the figure, please see Appendix references in S1 File. (TIF) [file pone.0126442.s002.tif]
