# Supplementary material for: Genetically Predicted Testosterone and Systemic Inflammation in Men: A Separate-Sample Mendelian Randomization Analysis in Older Chinese Men
Source: PLoS One. 2015 May 7;10(5):e0126442. doi: 10.1371/journal.pone.0126442 (PMC4423952; doi:10.1371/journal.pone.0126442)
Supplement: S1 File — (DOCX) [file pone.0126442.s003.docx]

**Appendix References**

1. Olson SH, Bandera EV, Orlow I (2007) Variants in estrogen biosynthesis genes, sex steroid hormone levels, and endometrial cancer: a HuGE review. Am J Epidemiol 165: 235-245.

2. Travis RC, Schumacher F, Hirschhorn JN, Kraft P, Allen NE, Albanes D, et al. (2009) CYP19A1 genetic variation in relation to prostate cancer risk and circulating sex hormone concentrations in men from the Breast and Prostate Cancer Cohort Consortium. Cancer Epidemiol Biomarkers Prev 18: 2734-2744.

3. Sowers MR, Wilson AL, Kardia SR, Chu J, Ferrell R (2006) Aromatase gene (CYP 19) polymorphisms and endogenous androgen concentrations in a multiracial/multiethnic, multisite study of women at midlife. Am J Med 119: S23-30.

4. Ahn J, Schumacher FR, Berndt SI, Pfeiffer R, Albanes D, Andriole GL, et al. (2009) Quantitative trait loci predicting circulating sex steroid hormones in men from the NCI-Breast and Prostate Cancer Cohort Consortium (BPC3). Hum Mol Genet 18: 3749-3757.

5. Chen YC, Kraft P, Bretsky P, Ketkar S, Hunter DJ, Albanes D, et al. (2007) Sequence variants of estrogen receptor beta and risk of prostate cancer in the National Cancer Institute Breast and Prostate Cancer Cohort Consortium. Cancer Epidemiol Biomarkers Prev 16: 1973-1981.
